# Dual NRF2 paralogs in Coho salmon and their antioxidant response element targets^☆^

**DOI:** 10.1016/j.redox.2016.07.001

**Published:** 2016-07-06

**Authors:** Richard Ramsden, Evan P. Gallagher

**Affiliations:** Department of Environmental and Occupational Health Sciences, University of Washington, Seattle, WA, USA

**Keywords:** Nuclear factor erythroid 2-like 2, Nrf2, Antioxidant response element, Coho salmon, Paralog

## Abstract

The transcription factor NFE2L2 (Nuclear Factor, Erythroid 2-Like 2, or NRF2) plays a key role in maintaining the redox state within cells. Characterization of this pathway has extended to fish, most notably zebrafish (*Danio rerio),* in which two paralogs of the transcription factor exist: Nrf2a, an activator, and Nrf2b, a negative regulator during embryogenesis. Only one ARE target has been thoroughly delineated in zebrafish, and this deviated from the canonical sequence derived from studies in mammals. In general, the mechanistic pathway has not been characterized in non-model aquatic organisms that are commonly exposed to environmental pollutants. The current study compares the zebrafish paralogs to those found in a non-model teleost, the ecologically important salmonid, *Oncorhnychus kisutch* (coho salmon). Two salmon paralogs, Nrf2A and -2B, described here were found to possess only slightly greater identity between one another (84% of amino acids) than to the singleton ortholog of the esocid *Esox lucius* (80–82%), the nearest non-salmonid outgroup. Unlike one of the zebrafish forms, each is a strong activating factor based on sequence homology and *in vitro* testing. To uncover functional target AREs in coho, promoter flanking sequences were isolated for five genes that protect cells against oxidative stress: heme oxygenase 1, peroxiredoxin 1, glutamate-cysteine ligase, and the glutathione *S*-transferases *pi* and *rho* (*hmox1, prdx1, gclc, gstp,* and *gstr*). All except *gstr* had functional elements and all fit the standard mammalian-derived canonical sequence, unlike the motif found in zebrafish *gstp*. Expression studies demonstrate the presence of both Nrf2 paralogs in multiple organs, although in differing ratios. Collectively, our findings extend the conservation of Nrf2 and the ARE to salmonids, and should help inform future work in teleosts on mechanisms of redox control, as well as responsiveness of this pathway and its downstream antioxidant gene targets to chemical exposures in the environment.

## Highlights

• Salmon possess dual paralogs of Nrf2 (Nfe2l2).
• The paralogs are disproportionately expressed among tissues.
• Antioxidant response elements in salmon follow canonical mammalian motifs.

## Graphical abstract

**Figure:**
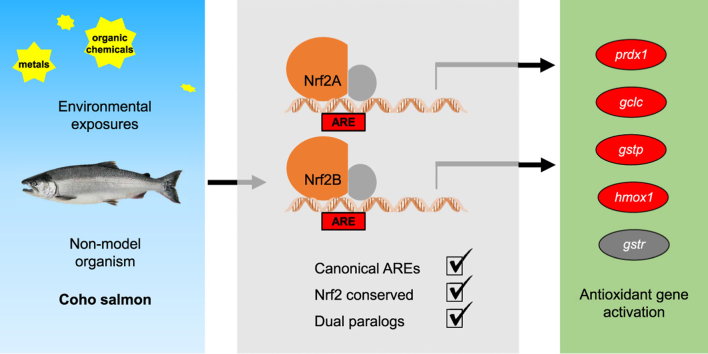

## 1 Introduction

The response of cellular homeostasis to electrophiles and reactive oxygen species (ROS) is regulated by NRF2 (NFE2L2), a basic leucine zipper (bZIP) transcription factor in the NFE2 (Nuclear Factor, Erythroid 2) family [36], and KEAP1 (Kelch-like ECH-associated 1), a substrate adapter protein for the E3 ubiquitin ligase complex which regulates its degradation [59]. At steady states, a homodimer of KEAP1 binds a single molecule of NRF2 facilitating its ubiquitination and proteolysis in the cytoplasm. Sulfhydryl groups of multiple cysteine residues within Keap1 become modified under conditions of oxidative stress perturbing its E3 ligase activity to disrupt the cycle of degradation and enabling NRF2 to translocate to the nucleus [see reviews [7], [48]]. Following translocation, NRF2 partners with other bZIP proteins, the small Mafs, to bind genomic antioxidant/electrophile response elements (ARE/EpREs) to activate the transcription of an array of cytoprotective genes, including those involved in maintaining redox status, xenobiotic metabolism, anti-inflammation, DNA repair, and others. Endogenous sources of ROS include electron loss from the mitochondrial transport chain while exogenous ones include chemicals that may be sorted into ten different types if grouped by structure [10] or placed into six different classes if separated by which KEAP1 cysteine is preferentially targeted [24].

Studies of the NRF2 pathway begun in human and rodent models, has also been extended to the zebrafish, *Danio rerio*. The latter work determined that the Nrf2-Keap1 system is conserved in zebrafish [23], that knockout of Nrf2 in this model organism increases susceptibility to oxidative insult just as it does in mice [37], [42], and that control of *gstp* (glutathione S-transferase pi) is mediated by Nrf2 through an ARE-like target similar to mouse [47], [13]. On the other hand, added complexities have also been described for fishes such as the existence of two Keap1 isoforms in many genera [31] and an extra Maf partner, tMaf, which is exclusive to teleosts [49]. Furthermore, recent studies have discovered two Nrf2 paralogs in zebrafish: Nrf2a, which is a strong activator of transcription and analogous to mammalian orthologs, and Nrf2b, which may act as a repressor of transcription during development [53], [9].

Investigations into cellular protective responses to oxidative stress in ecological (non-model) teleost species has been limited relative to studies in rodents and cell lines, and are often focused on molecular and cellular endpoints such as modulation of mRNA, enzymatic activation, DNA damage, *etc*. [11]. The underlying molecular mechanisms are rarely investigated in detail. The present study examines Nrf2 of coho salmon (*Oncorhynchus kisutch).* Coho are one of several ecologically important species of Pacific salmon that become exposed during residence or migration to environmental pollutants which have been linked to their population decline in the western United States [28], [41]. A key component of this phenomenon is sublethal chemical-mediated neurological injury, specifically damage to the peripheral nervous system including loss of olfactory function. Reduced olfaction can impair predator detection, prey selection, reproductive timing, imprinting, and homing behaviors (reviewed in [52]). Maintaining cellular antioxidant defenses in olfactory and other tissues in response to chemical injury is mediated in large part by NRF2-activated pathways [56].

The current study describes two Nrf2 paralogs from coho, and the regulatory promoters of five important antioxidant defense genes, including heme oxygenase 1, peroxiredoxin-1, glutamate-cysteine ligase catalytic subunit, and glutathione S-transferases *rho* and *pi* (*hmox1, prdx1, gclc, gstp,* and *gstr*). Expression of these newly described paralogs is investigated within various coho tissues and functional ARE-specific activation is demonstrated against multiple endogenous targets. This study may inform future mechanistic work on teleosts in the area of comparative biochemistry and aquatic toxicology.

## 2 Methods

### 2.1 Isolation of coho gene promoter fragments

All animal welfare and experimental procedures were carried out in accordance with the University of Washington Institutional Animal Care and Use Committee (IACUC) guidelines. DNA was isolated from liver of a one-year-old hatchery-reared coho salmon (Issaquah salmon hatchery, Washington Department of Fish and Wildlife, Issaquah WA) by proteinase K digest (2 mg/ml in 50 mM Tris-HCl, 50 mM EDTA, 1% SDS, 10 mM NaCl, pH 8) at 37° followed by phenol extraction and precipitation in isopropanol using standard techniques. Following a described method [54], genomic DNA was variously digested (*Bam*HI, *Bst*BI, *Bgl*II, *Eco*RI, *Hind*III, *Nco*I, *Nde*I, *Nhe*I, *Spe*I, or *Xba*I) then circularized by ligation with T4 DNA ligase (NEB) overnight. After precipitation, 10 μM Exo-Resistant Random Primers (Thermo Fisher Scientific) were added to 5% of the volume of each library in the presence of 1 mM dNTPs, heated to 95° for 10 min, then cooled before the addition of *Ø*29 DNA polymerase (Thermo Fisher Scientific). Rolling circular amplification continued for 16 h at 22°. These libraries served as templates for inverse PCR and genomic DNA for standard PCR. DNA oligonucleotide primers used were designed against published sequence of coho or nearest relative for *gclc*, *gstp*, *gstr*, *hmox1*, and *prdx1* then amplified using Phusion DNA polymerase (NEB) or GemTaq (MGQuest) using standard protocols. Products were isolated using agarose gel electrophoresis, the amplicons purified (GeneJET, Thermo Scientific) and sequenced (Eurofins). Primers based upon these sequences were used to amplify flanking regions for direct cloning into the luciferase reporter pGL3 (firefly luciferase, Promega). Site-directed mutagenesis of putative AREs was done using standard methods (QuikChange kit, Agilent Technologies) and confirmed by sequencing. For primers used in this study, see Supplemental Table 1. The sequence of all clones is available in Supplemental Table 2.

### 2.2 Isolation of Nrf2 isoforms

Total RNA from 4 day old zebrafish larvae (strain EKW) was isolated using standard methods (TRIzol®, Invitrogen™) and cDNA synthesized using oligo d(T) and SuperScript II® (Invitrogen™). This served as template for amplification by Phusion DNA polymerase with primers bases upon published coding sequence of Nfe2l2a and Nfe2l2b. The amplicons were cloned into pcDNA (Invitrogen™). The resulting clones, pcDNAzNrf2a and -2b, coding regions were confirmed by sequencing. RNA from an adult coho liver was extracted, cDNA obtained, then treated similarly before PCR amplification. Primers were designed against flanking sequences from two salmon forms found in databases: GenBank FR905794, locus tag GSONMT00046250001 (*O. mykiss*) with NM_001139807 (*S. salar*) for Nrf2A and GenBank HG973522, locus tag GSONMT00008906001 (*O. mykiss*) with BT059007 (*S. salar*) for Nrf2B. Amplicons cloned into expression vectors designated pcDNAcohoNrf2A and -2B were confirmed through sequencing.

### 2.3 Cell culture and assays

Zebrafish cell line ZEM2S was obtained from ATCC® and grown in Leibovitz's L-15 media (ATCC®) supplemented with 10% fetal bovine serum (Atlanta Biologicals, Atlanta, GA) and grown at 28–29.5°. Cells were seeded into white 96 well microplates at 60–65,000 cells/well in 75 μl media before transfection 24 h later. Each treatment included DNA mixes of 24 ng pcDNA expression vectors [(6 ng pcDNA-*x*Nrf2*x* + 18 ng empty pcDNA) or 24 ng pcDNA] with 50 ng pGL3 reporters plus 25 ng pRL-CMV (renilla luciferase loading control, Promega) prepared in 100 μl Opti-MEM (Gibco) in the presence of 0.3 μl X-tremeGENE 9 (transfection reagent, Roche). Negative controls included either all empty vectors or no DNA, positive control was pW1 EpRE MODLUC EGFP vector (provided by Dr Michael Carvan, University Wisconsin-Milwaukee). After a 30–45 m incubation at room temperature, 10 μl of mix was added per duplicate well. Microplates were cultured 20 h then medium removed and replaced with 35 μl phosphate buffered saline and an equal volume of firefly luciferase reagent (Dual-Glo Luciferase kit, Promega) added then assayed. For normalization, an equal volume of renilla luciferase reagent was subsequently added then assayed after a short incubation. Luminescence was measured on a luminometer (PlateLumino, Stratec Biomedical). Results shown are the mean of values derived from three replicate experiments. Preliminary reporter tests were performed titrating Nrf2 expression vectors to insure luciferase values were submaximal and in a linear range (data not shown). Final transfection efficiency was 1% and all measurements were assessed between 13 and 24 cell passages.

### 2.4 Analysis of tissue specific coho Nrf2 paralog expression

Five one-year-old coho reared in fresh water were provided by NOAA Northwest Fisheries Science Center (Seattle, WA) and euthanized using Tricaine. Seven tissues were harvested and initially stored in RNAlater® (Ambion): brain, gill, gonad (testes or ovary), heart, kidney, liver, and olfactory rosettes. Upon processing, the RNAlater® was removed, replaced with TRIzol™ (Invitrogen), and tissues ruptured on a TissueLyser (Qiagen) using 5 mm steel beads at 50 Hz for 5 min. Following extraction and precipitation as in the manufacturer's protocol, RNA pellets were resuspended in water and quantified using a NanoDrop ND-1000 (Thermo Scientific). Equal amounts of RNA (400 ng) were treated with DNase I (Invitrogen) and cDNA synthesized using iScript™ Reverse Transcription kit (Bio-Rad). After 5-fold dilutions in water, quantitative PCR analysis was performed using SsoAdvanced™ Universal SYBR® Green Supermix (Bio-Rad) on an iQ5 cycler (Bio-Rad) using a standard curve method [5] against reference genes *eef1a1a* and *eef1a1b* [1]. Before assays were conducted, primers were tested to optimize annealing temperatures and to insure single product formation whose identities were confirmed through sequencing. Primer sequence for Nrf2 paralogs and reference genes are listed in Supplemental Data. After initial denaturation at 95°, 2 m, assay parameters were [95° 10 s, 60–62° 20 s, 72° 15 s] 35 cycles followed by a melt curve.

## 3 Results

### 3.1 Salmonids possess two Nrf2 paralogs

**Fig. 1:**
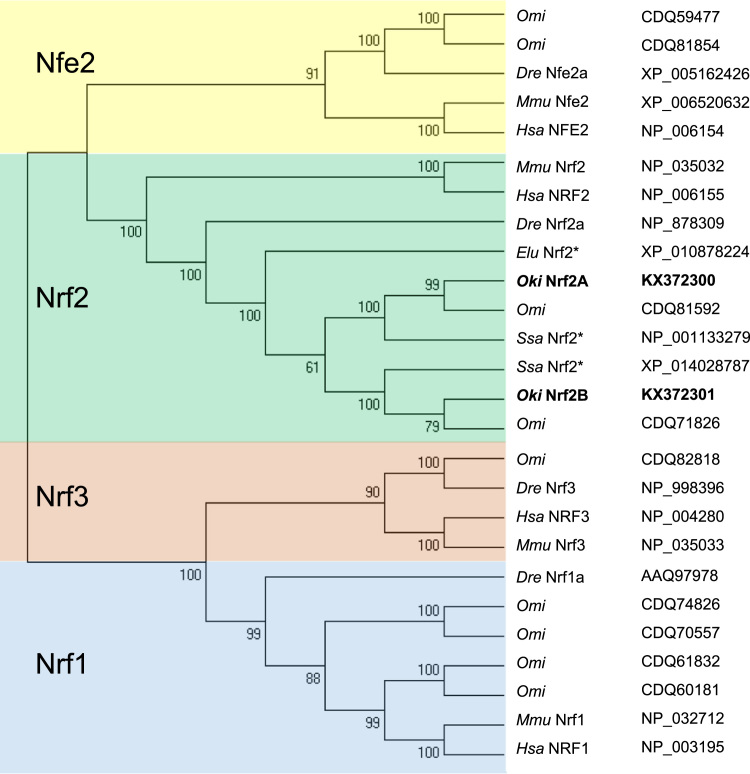
Phylogenetic tree reveals relational grouping of Nrf-like proteins from rainbow trout. The tree was constructed using MEGA6 and its maximum likelihood method with previously curated protein sequences for human, mouse, and zebrafish deposited in GenBank and unknown syntenically related protein sequences from the rainbow trout genome database (http://www.genoscope.cns.fr/trout/) with additional sequences for pike (GenBank) and the coho paralogs (this study). Branch numbers represent frequencies (%) of presented tree topology after bootstrapping (500 iterations). *Mmu*, mouse; *Hsa*, human; *Dre*, zebrafish; *Omi*, rainbow trout; *Oki*, coho; *Elu*, northern pike. Paralogs described in this study, bold. *Predicted identity in NCBI database.

A query of human NRF2 amino acid sequence (GenBank NP_006155) using the BLAST tool against the National Center for Biotechnology Information (NCBI) database returned two coding sequences with high similarity among salmonids, including rainbow trout, *Oncorhynchus mykiss* (CDQ81592 and CDQ71826) and Atlantic salmon, *Salmo salar* (NP_001133279 and XP_014028787). Although each salmon pair is 84% identical at the amino acid level, all sequences demonstrate an overall 47% identity to human and are similarly sized (611/612 a.a. and 605 a.a., respectively). To detect the same pair in coho salmon, primers were designed against the outer untranslated regions of the two putative Nrf2 forms from Atlantic salmon and rainbow trout and used to amplify total cDNA from coho liver using standard PCR. The resulting sequence from these amplicons revealed the same Nrf2 pair in coho. To establish that no more than two Nrf2 forms are likely to exist in coho, the cDNA sequence of one, CDQ71826 (GSONMG00008906001), was used to capture all Nrf-related syntenic genes available from the rainbow trout genome database (http://www.genoscope.cns.fr/trout/) [2]. Those obtained included GenBank accessions CDQ59477, CDQ81854, CDQ81592, CDQ71826, CDQ82818, CDQ74826, CDQ70557, CDQ61832, and CDQ60181. Phylogenic analysis of these along with curated Nrf-related proteins from zebrafish, mouse, and human orthologs using MEGA software (version 6) [50] indicated as many as nine such genes in the trout, including four Nrf1, one Nrf3, two Nfe2, and two Nrf2 forms (Fig. 1). A second form of Nrf3 found in the search of the NCBI database may exist for Atlantic salmon (XM_014142932). It is therefore possible that as many as ten related Nrf-like salmonid genes may exist. The two distinct Nrf2 proteins are designated in the present study as Nfe2l2A or *Oki*Nrf2A for coho (rainbow trout CDQ81592, Atlantic salmon NP_001133279) and Nfe2l2B or *Oki*Nrf2B for coho (rainbow trout CDQ71826, Atlantic salmon XP_014028787). For these salmon species, within each A or B form there is 96–99% identity, while this drops to 84% identity between forms. The Nrf2 gene sequences have been deposited with GenBank (accessions KX372300 and KX372301 for *Oki*Nrf2A and -2B, respectively).

### 3.2 Divergence of coho NRf2 paralogs and northern pike ortholog

**Fig. 2:**
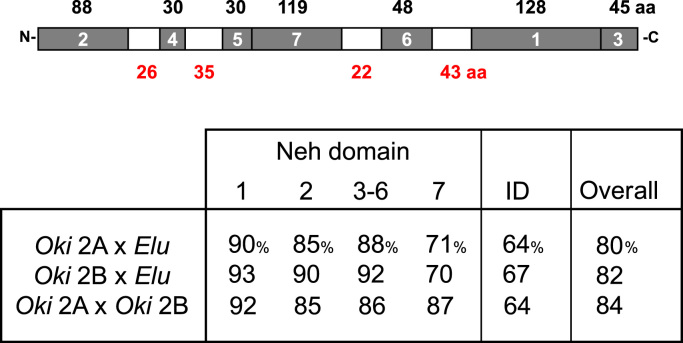
Shared identity between the Nrf2 paralogs of coho and northern pike indicates divergence within Neh7 domain. Coho paralogs (*Oki*) and the pike (*Elu*) ortholog share similarly high identities (85–92%) in canonical Neh domains 1–6 and similarly lower identities in interdomain (ID) regions (64–67%). High identity is maintained between the coho forms within Neh7 while diverging from the pike ortholog. The schematic represents *Oki* Nrf2A with Neh regions numbered and amino acid count of domains and ID regions indicated above and below, respectively.

The order Salmoniformes underwent a whole genome duplication (WGD) 95 MYA and speciation 50 MYA [2], [33]. This group shares a common lineage with the order Esociformes represented by northern pike, *Esox lucius*, the nearest non-polyploid sister outgroup [14], [30]. Comparison of the pike Nrf2 ortholog as the outgroup (NCBI reference sequence XP_010878224) to the coho paralogs might suggest how the duplicated genes may have evolved through time. The average ratio (ω) of changes to the triplet code that are nonsynonymous (i.e. those that alter the amino acid, dN) to synonymous ones (those that do not alter, dS) for the coho paralogs against pike is <1 (ω = 0.21 [2A]; ω = 0.16 [2B]) indicating negative or purifying selection (dN=0.1001, dS=0.4790 [2A]; dN=0.0905, dS=0.5684 [2B]) as determined by SNAP (http://www.hiv.lanl.gov/content/sequence/SNAP/SNAP.html) [26]. In terms of overall amino acid identity, salmon paralogs 2A and 2B remain more similar to one another (84%) than either is to pike (79% and 81%, respectively). Identity is higher among canonical domains Neh1–6 (>85%) than inter-domain regions (<67%), reflective of greater evolutionary constraint on functional regions. Furthermore, sequence identity values are equivalent whether comparing 2A with 2B or either against pike, whether in Neh1–6 or the interdomain regions, with the exception of the Neh7 domain (Fig. 2). There, the paralogs 2A and 2B still maintain an identity to one another, just as high as in the other domains (87%), while each has diverged from the pike ortholog (~70% identity).

### 3.3 Tissue-specific Nrf2 paralog expression

**Fig. 3:**
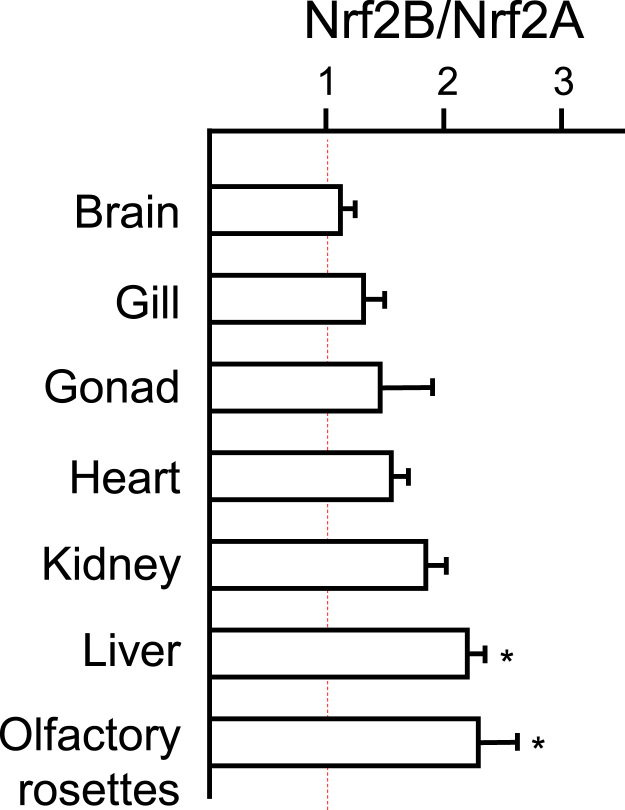
Coho Nrf2B mRNA is more abundant than Nrf2A in various tissues. Relative mRNA levels of the coho Nrf2 paralogs as determined by quantitative PCR of cDNAs, normalized to reference gene *eef1a-B*, and the ratio *Nrf2B:Nrf2A* plotted. Dashed line: ratio = 1 or equal levels of expression. *Indicates significant differences in NRF2 paralog expression at p < 0.05, One way ANOVA and Dunnett’s multiple comparisons test. *n* = 5.

The proportional expression of both salmon paralogs was examined in seven tissues (brain, gill, gonad, kidney, heart, liver, and olfactory rosette) from five animals using real-time PCR. In each tissue, Nrf2B was slightly more abundant than Nrf2A, though not rising to a level of statistical significance (p > 0.05), except in liver and olfactory rosettes where Nrf2B levels were 2.2-fold greater (p < 0.05) than observed for Nrf2A (Fig. 3). This trend was similar when data was normalized to reference gene *eef1a1b* (Fig. 3) or a second reference gene, *eef1a1a* (data not shown). It should be noted that the relative abundance refers to mRNA levels only, and may not necessarily be reflective of post-translationally state of the messages.

### 3.4 Coho Nrf2 activation of endogenous targets *in vitro* is ARE-dependent

**Fig. 4:**
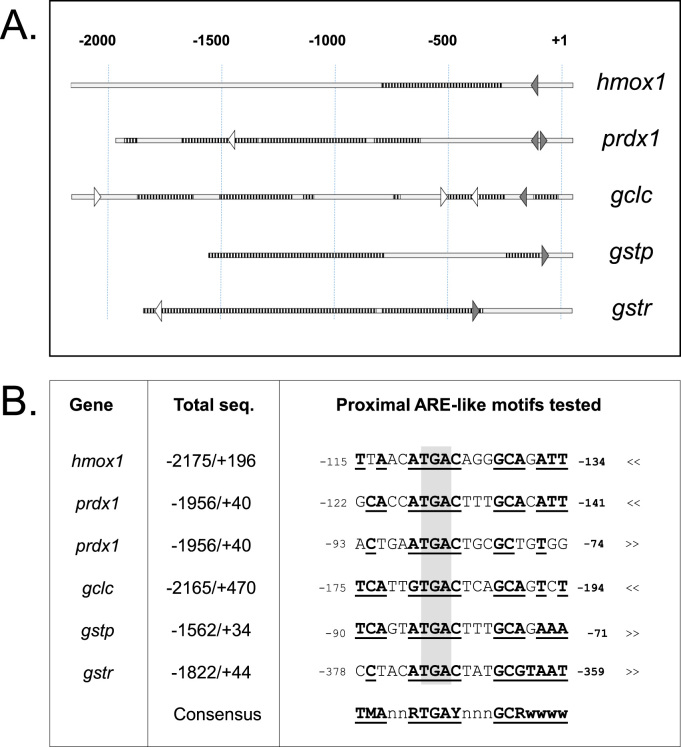
Proximal promoter sequences of coho contain putative ARE-like elements. (A) Schematic of proximal sequences from coho tested in reporter assays. Striated bars indicate regions of repetitive and open bars non-repetitive sequence. Triangles represent matches to core ARE (RTGAYNNNGC), right- or left-facing indicate forward or reverse orientation, respectively, and those filled represent elements examined in this study. Numbering is approximate to putative transcription start sites based on cDNA data available for *Salmo salar* [GenBank BT047880 (*hmox1*), NM_001141386 (*prdx1*), BT058249 (*gstp*), BT057296 (*gstr*); CSSEST2 database, id SS2U041775 (*gclc*)]. (B) Alignment of proximal coho ARE motifs with mammalian consensus. Putative ARE elements from coho genes examined in this study. Bases in agreement to consensus sequence underscored and bold. Numbers represent approximate position relative to transcription start in forward (>>) or reverse (<<) orientation. Shaded box indicates mutation target: TGA→ATT (*hmox*1 and *gclc*) or TGA→CCC (*prdx1, gstp, gstr*). Region of flanking sequence tested in pGL3 luciferase reporter constructs, middle column. Consensus: (M = A or C, R = A or G, Y = C or T, W = A or T) [57].

A prime function of the transcription factor Nrf2 is to bind an ARE and activate transcription from a *cis* promoter. Upstream promoter regions of genes known from other organisms to be controlled by Nrf2, *prdx1, gstp, hmox1,* and *gclc,* were isolated by polymerase amplification from coho genomic DNA. The *gstr* promoter, a gene unique to teleosts of unknown responsiveness to Nrf2, was also isolated. Between 1500 and 2200 bp DNA sequence was determined for each (Supplemental file). About half of the flanking sequence obtained for *gstp, gclc,* and *prdx1* was found to be composed of interspersed repeat elements while *hmox1* contained less (24%) and *gstr* much more (80%), as determined by the Repeat Masker tool by GRASP (Genomic Research on Atlantic Salmon Project, http://grasp.mbb.sfu.ca/GRASPRepetitive.html). Sequences searched for a core ARE sequence RTGAYNNNGC using fuzznuc from EMBOSS (European Molecular Biology Open Software Suite) (http://www.bioinformatics.nl/cgi-bin/emboss/fuzznuc) found at least one match in each gene within 380 bp of the putative start of transcription (Fig. 4A).

**Fig. 5:**
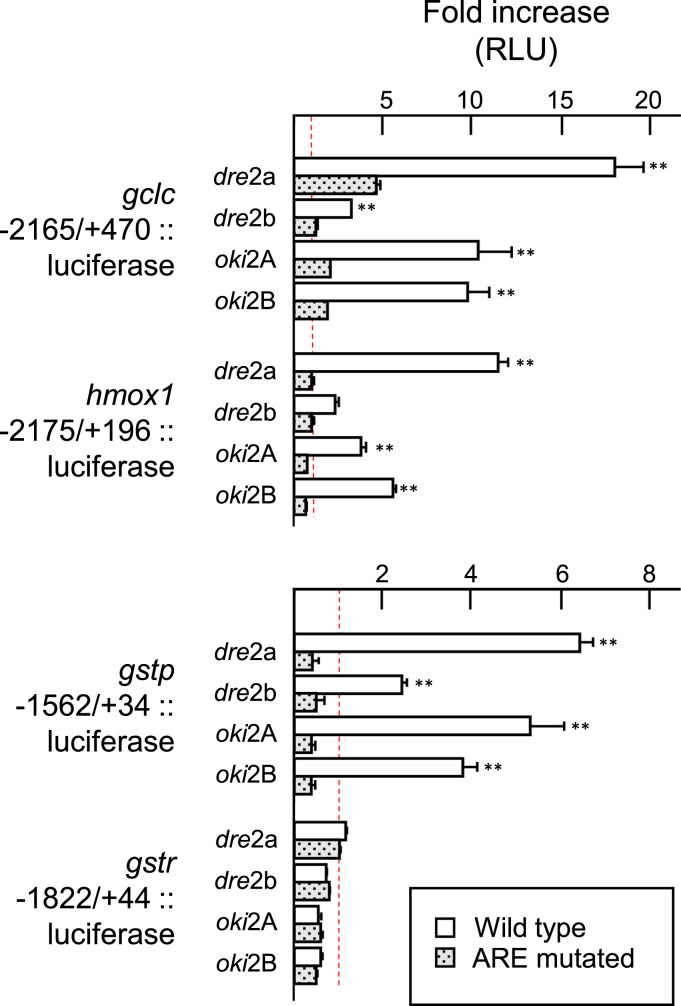
The activation of coho gene reporters *gclc*, *hmox1*, and *gstp* by Nrf2 is dependent on an intact ARE. Promoter gene fragments from coho *gclc, hmox1, gstp,* and *gstr* were inserted into the luciferase reporter plasmid pGL3 (whole bars) or with putative AREs mutated (stippled bars) and co-transfected along with CMV-driven cDNAs of zebrafish paralogs Nrf2a or -2b (*dre*2a or 2b), coho paralogs Nrf2A or -2B (*oki*2A or -2B) or null vector (pcDNA3) into the zebrafish embryonic cell line ZEM2S. Fold changes reflect the ratio of each reporter co-transfected with Nrf2-expressing vector against the same reporter with the null vector. A third vector, pRL-CMV, was also added to normalize transfection efficiency. All values derive from duplicate samples in each of three independent transfections. Dashed line delineates 1 or no change in expression relative to null vector. RLU, relative light units. **Indicates differences in reporter activity relative to respective wild type or mutant, p < 0.01, two-way ANOVA and Bonferroni post-test.

**Fig. 6:**
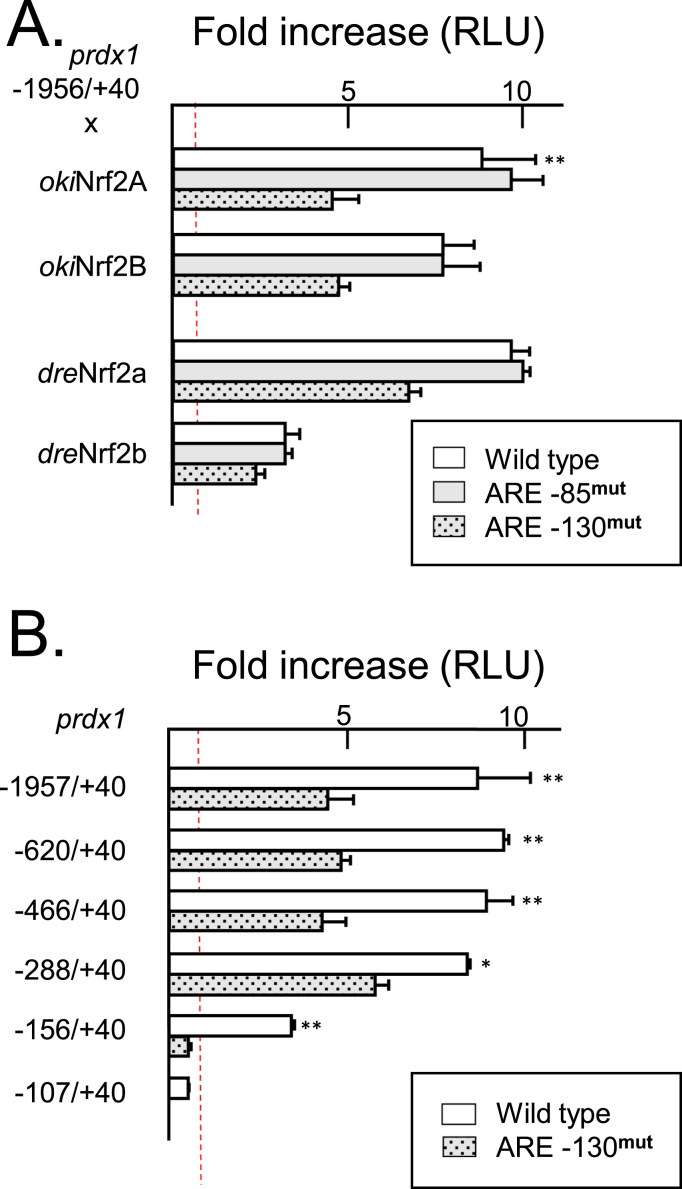
Deletion and mutation analysis of *prdx1* promoter reveals its activation may be partially dependent on ARE-130 and other unknown elements. (A) A genomic DNA fragment from coho *prdx1* −1956/+40 was inserted into the luciferase reporter plasmid pGL3 as wild type (white bars) or with putative AREs mutated at -85 (gray bars) or -130 (stippled) and co-transfected with vectors expressing zebrafish or coho paralogs of Nrf2 as noted then compared to levels achieved with empty vector, pcDNA3, to determine fold increase. (B) Only an abbreviated *prdx1* promoter indicates activation wholly dependent upon ARE-130. Serial deletion analysis of the *prdx1* promoter with the -130 ARE intact (wild type, white bar) or mutated (stippled) and co-transfected with a vector expressing coho Nrf2A. Fold increases obtained and calculated as above. Dashed line delineates 1 or no increase each scale. Differences in reporter activity relative to respective wild type or mutant, *p < 0.05, **p < 0.01, two-way ANOVA and Bonferroni post-test.

The *gstp, gstr*, *gclc, hmox1,* and *prdx1* promoters were cloned into luciferase reporter vectors to test their overall ability for activation by coho and zebrafish forms of Nrf2. All of the promoters tested, with the exception of *gstr,* were activated strongly by coho Nrf2A and Nrf2B as well as zebrafish Nrf2a, but only weakly by the zebrafish paralog Nrf2b. A complementary group of vectors was also created with the most proximal predicted ARE motifs mutated (Fig. 4B) to test if activation is mediated through these elements. Activation was drastically reduced or abolished for *hmox1, gclc,* and *gstp* when these proximal ARE-like sites were mutated implicating these as major control elements (Fig. 5). Two proximal ARE-like targets were tested for *prdx1*. Activation of the maximum length tested (1956 bp) was not impaired by the mutation of the ARE-like sequence at position -85 and reduced only by half upon mutation of the ARE-like sequence at position -130 (Fig. 6A). Serial deletion testing of this promoter showed a similar pattern down to position -288 (Fig. 6B). Deletion down to position -156 diminished activation for the wild-type promoter and the additional mutation of the -130 ARE eliminated activation completely (Fig. 6B and data not shown).

## 4 Discussion

**Fig. 7:**
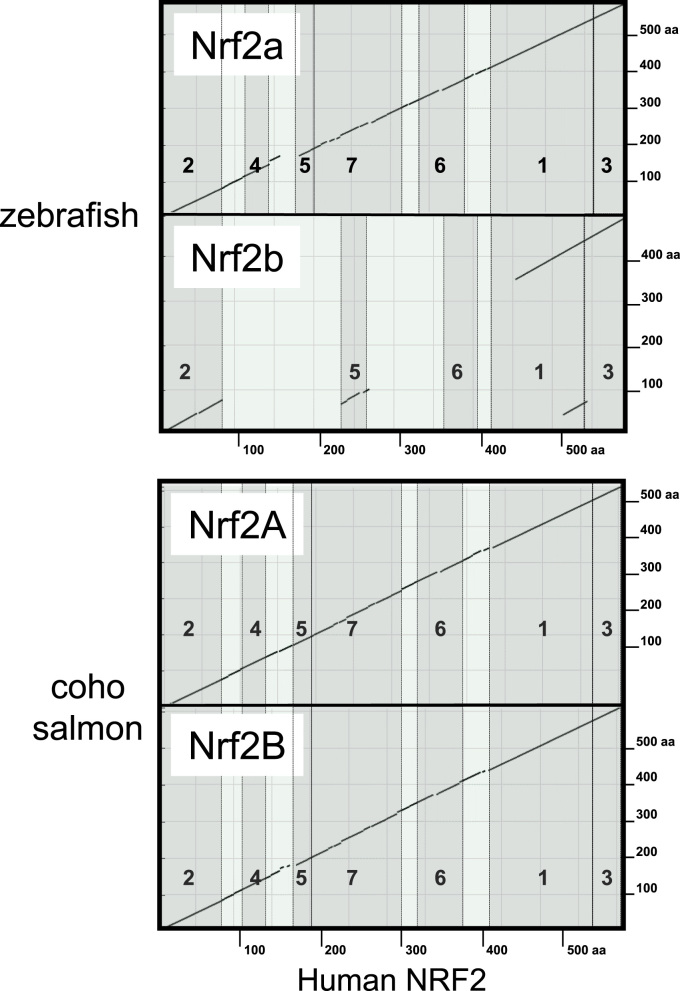
Protein sequence alignment of dual zebrafish paralogs and salmon paralogs with the human NRF2 ortholog. Dot matrices indicate regions of similarity based upon the BLAST algorithm. Shaded bands with numbers highlight Neh domains.

This report describes the occurrence of dual paralogs of the critical transcription factor Nrf2 in coho salmon. These forms share an overall 47% identity to the human ortholog, NRF2. A similar comparison of the two zebrafish forms shows *Dre* Nrf2a to have 45% identity to human and the abbreviated form, *Dre* Nrf2b, only 25% identity (Fig. 7). Seven areas of homology have been noted between human NRF2 and the chicken ortholog (ECH): Neh1–7 (Nrf2-ECH homology) domains [15], (reviewed in [51]). Critical features within these regions are also highly conserved in *Oki*Nrf2A and *-*2B.

**Fig. 8:**
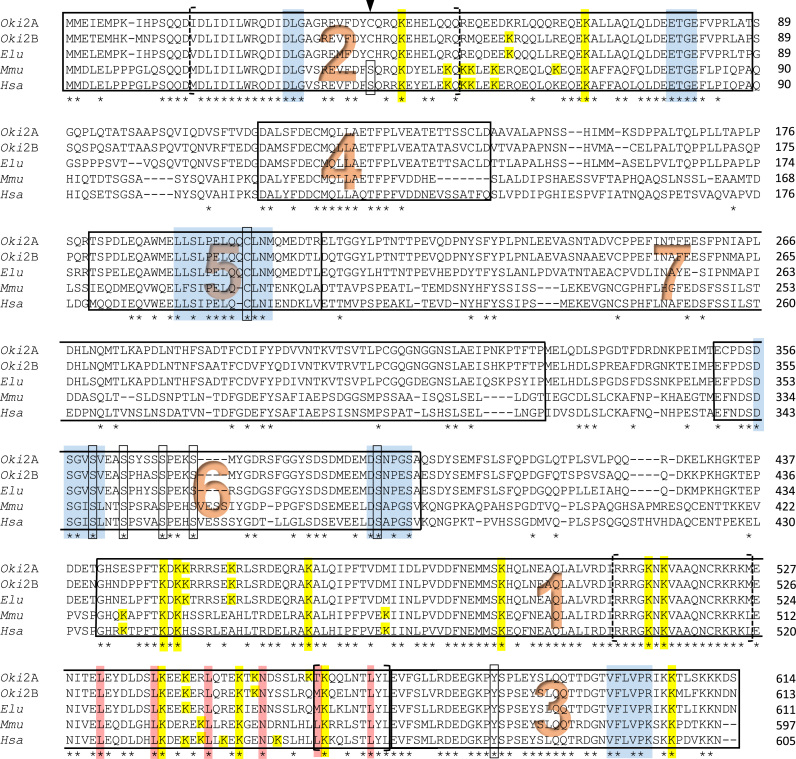
Alignment of salmon Nrf2 forms with human and mouse orthologs reveal identity at critical residues. Neh domains 1–6 delineation follows previous work in zebrafish with the addition of Neh7 [53], [55]. Neh1: DNA-binding and heterodimerization domain. Leucine zipper [36] (red boxes), NLS (dashed brackets) and NES (solid brackets) (Jain, Bloom et al., 2005), potential lysine targets of acetylation (yellow boxes) [46]. Neh2: Keap1-mediated degradation domain. Keap1-contacting motifs DLG and ETGE (blue boxes) [35], [23], DLGex (dashed brackets) (Fukutomi, Takagi et al., 2014), lysine targets for ubiquitination (yellow boxes) [59], PKC phosphorylation site (box and arrow) [12], [40]. Neh3: Transactivating motif (blue box) [39], tyrosine targeted by Fyn kinase (open box) [17], activating acetylation site (yellow box) [20]. Neh4 and Neh5: Activating domains [19], [22], [34]. NES (blue box) with redox-sensing cysteine (open box). Neh6: Redox-insensitive degron. β-TrCP recognition sites (blue boxes), putative GSK-3 phosphorylation sites (open boxes) [3]. Neh7: RXRα-interacting region [55]. The coho salmon (*Oki*) forms of Nrf2 described in this study were aligned against mouse (GenBank accession number NP_035032), human (NP_006155), and pike (XP_010878224) using Clustal Omega [45]. (For interpretation of the references to color in this figure legend, the reader is referred to the web version of this article.)

The amino terminal, Neh2, is the domain that interacts with the negative regulator Keap1 which directs ubiquitylation there under homeostatic conditions. The DLG and the ETGE docking motifs [35], [23] are present in both salmon Nrf2 paralogs, and the extended motif, DLGex, [6] is highly conserved [(Fig. 8) here and for the proceeding section]. Seven lysines in Neh2 of human NRF2 can act as target sites for ubiquitination [59]; only three such sites are found in the coho forms of this domain though only one is sufficient to act as target of the Cul3 ubiquitin ligase to promote Nrf2 degradation by the proteasome [59]. A serine at position 40 (human numbering) is found in other model vertebrate orthologs including those from human, mouse, rat, chicken, and zebrafish. This site has been described as critical for phosphorylation by protein kinase C (PKC) to mediate release of Nrf2 from Keap1, leading to its stabilization, nuclear translocation, and thus activation of ARE-regulating genes [12], [40]. A cysteine occupies the equivalent position in salmon forms and no other canonical PKC targets (S/T)*X*(R/K) appear in the Keap1-interaction domain Neh2 of *Oki*Nrf2A or -2B. In teleosts, this site may be a serine (zebrafish, herring, tetra), cysteine (salmon and pike) or asparagine (pufferfish, guppy, medaka, tilapia, and others) (Suppl. Fig. 9). While it has been suggested that a serine in mammalian forms is a target of phosphorylation by PKC, others have found that Ser40 phosphorylation does not affect interaction [6]. The variant sequence found in the coho forms would support the latter view.

Neh1 is another key feature of Nrf2 transcription factors. Beginning at Gly434 (human, Gly441 *Oki*Nrf2A), this domain includes a basic region that can bind to DNA directly as well as a leucine zipper (bZIP) [36] to interface with DNA binding partners like small Maf proteins, which then recognize the regulatory genomic AREs. As the basic region is all but identical between mammalian and salmon forms, the DNA half site for Nrf2 recognition could be anticipated to be similar. As with other DNA binding factors, this region may also act as a nuclear localization signal (NLS) [16]. A nuclear export signal (NES) has also been located in this domain [16]. Only coho Nrf2B fully matches for the leucine-rich NES consensus ϕ-*X*_**2–3**_-ϕ-*X*_**2–3**_-ϕ-*X*-ϕ (ϕ=hydrophobic residues L, I, V, F, M; *X*=any amino acid) [27] while coho Nrf2A has a tyrosine at the first position. Multiple lysine residues scattered throughout this domain [18 (human), 16 (coho 2A), 15 (coho 2B)] act as redundant target sites that may augment transcriptionally activity upon acetylation [46].

Besides the DNA-binding and Keap1-regulating domains, domains Neh3, -4, and -5 are involved in transactivation by the transcription factor. While these are the regions of greatest differences with the Nrf2 paralogs of zebrafish – Neh4 is wholly absent in its Nrf2b form [53] – the paralogs of salmon seem to retain all core elements when aligned with human/mouse forms. One element in Neh3, the VFLVPK motif (position 591–6, human) that has been described as necessary for transactivation is also present in the salmon (VFLVPR, coho 598–603) [39]. Tyr576 human/ Tyr568 mouse, described as a phosphorylation target of Fyn kinase and essential for nuclear export [17] is also present at the analogous position in both salmon forms although absent in zebrafish Nrf2a. Lys599 human/Lys591 mouse that once acetylated promote nuclear localization and activation [20] is likewise present in both *Oki*Nrf2A and -2B (Lys606 and Lys605, respectively).

Core regions of Neh4 and -5 identity are also shared between mammalian and salmon forms. These domains have been found to cooperatively bind CBP (CREB binding protein) as well as RAC3 (Receptor-associated coactivator 3) to facilitate gene activation [19], [22] or to Kap1 (KRAB-associated protein 1) to maximize transcription [34] in mammalian cell culture. A redox-sensing NES in Neh5 has also been described using similar *in vitro* methods [32] in which export to the cytoplasm under homeostatic conditions was interrupted by prototypic inducers DEM (diethyl maleate), H_2_O_2_, and tBHQ (tert-butylhydroquinone). The key redox-sensing cysteine residue within the canonical spacing of the hydrophobic residues is also found in the protein sequence of the both *Oki*Nrf2A and -2B.

The final two domains of Nrf2 includes a redox-insensitive degron in Neh6. The proline-directed serine/threonine kinase GSK-3 (glycogen synthase kinase-3) is reported to phosphorylate the DSGISL motif which then is recognized by the E3 ligase adapter β-TrCP (beta-transducin repeat containing protein) leading to ubiquitination by Cullin1/Rbx1 and Nrf2 degradation [3]. This mechanism can be expected to operate on the coho paralogs as well. β-TrCP recognizes the phosphorylated DpSGIpSL through interaction with hydrophobic residues leucine and isoleucine [4]; hydrophobic residue valine occupies the same positions in the salmon forms and may be expected to act similarly (DpSGVpSV). Proximal to the DSGISL motif is the ubiquitin-targeted lysine residue (12, 17, or 11 residues away, mouse, human, or coho, respectively), while distal is an array of serines that may act as the priming phosphorylation sites allowing recognition by GSK-3 [4]; *Oki*Nrf2B has a pair of these serine-proline sites arranged as in the mouse and human forms, whereas *Oki*Nrf2A has just one. The recently described Neh7 domain from mouse and human Nrf2 has been shown to directly interact with RXRα to inhibit activation [55]; only scattered identities are found in this region with the salmon forms.

The existence of two salmonid paralogs is presumably the result of the whole genome duplication event that occurred in that group of fishes after divergence from a diploid species that was also ancestral to northern pike. Within the canonical domains Neh1-6, homologies between the coho paralogs is just as high between themselves as with the pike ortholog of the Esocid outgroup. An exception was found in the RXRα-binding Neh7 domain where salmon paralogs retain high homology but diverge from pike. Although the reason for this is unclear, it does imply that this region may have added functional importance in salmonids.

The evolutionary importance of dual paralogs of Nrf2 is open to speculation. The genomes of salmonids have been reverting back to diploidy since the ancestral WGD event. In the analysis of the genome of rainbow trout (*Oncorhynchus mykiss*), it is reported that about half (48%) of the genes remain doubled while the rest (52%) have become singletons [2]. In coho, the doubled Nrf2 genes are still retained. Divergence of function seems unlikely based on the conservation of critical residues within the canonical Neh domains of both forms. Retention of function for both paralogs, i.e. the lack of positive diversifying function, is also indicated by the low dN/dS ratios <1 [58], consistent with a larger study of duplicated genes in Atlantic Salmon [30]. The subfunctionalization of Nrf2 forms as seen in zebrafish [53] developed over a much greater time scale: 320-350 MYA since the original WGD event in teleosts against 95 MYA for the salmonid WGD [8]. The negative selection of Nrf2 paralogs in salmon may be influenced by the large number of (and possibly duplicated) interacting partners required to co-evolve. Less restrictive paths to diverging function might come via spatial and temporal regulatory elements [18]. Altered *cis*-elements could change relative expression of the paralogs, as many as ten Nrf-like family members in salmon might compete for AREs to alter expression, and duplicated targeted genes themselves could freely gain or lose regulatory elements that develop independent expression profiles.

Functionality of coho Nrf2 paralogs were tested *in vitro* against putative coho target gene fragments in cell line ZEM2S in parallel with the zebrafish forms. Four of the genes tested included *gstp, gclc, hmox1,* and *prdx1,* whose induction by the electrophile cadmium had previously been shown to be dependent on Nrf2a in zebrafish [56]. The examination of each putative promoter region, from 1560 to 2175 bp long, found at least one match with the central region of the mammalian ARE consensus (RTGAYNNNGC) [57] (Fig. 4A). Each native gene reporter except *gstr* was strongly activated in the presence of *Oki*Nrf2A and -2B. Activation was abolished when mutations were introduced to the most proximal elements in *hmox1, gstp,* and *gclc* (Fig. 5) indicating that these may be functional regulatory AREs. Two potential ARE sites located at -85 and -130 were tested in the *prdx1* promoter: mutations within ARE-130 reduced activation only partially while mutation within -85 had no effect at all (Fig. 6A). Only upon deletion of upstream sequences beyond -157 was activation completely abolished by the mutated -130 ARE (Fig. 6B). These data suggest that there may be a functional ARE in the promoter of coho *prdx1* at -130 and unknown element(s) may exist upstream that might promote or stabilize ARE/Nrf2 binding. It should also be noted that the basal strength of all promoters examined varied over 30-fold with *prdx1* being the highest and *gclc* the lowest though the latter had the highest fold induction (Supplementary. Fig. 10).

An additional promoter was tested from *gstr*, a *rho* class GST unique to fishes and some other aquatic organisms [25]. In some species, these enzymes rapidly conjugate reactive α, β-unsaturated aldehydes such as 4-hydroxynonenal generated during lipid peroxidation [44]. Accordingly, GST rho may constitute an important route of cellular protection during oxidative stress which might otherwise lead to the formation of covalent adducts of these reactive intermediates with sulfhydryl groups of glutathione, the cysteine, lysine or histidine residues of proteins, and nucleophilic sites of nucleic acids [21], [43]. Fish may be particularly vulnerable to cellular oxidative damage due to high levels of polyunsaturated fatty acids that are susceptible to peroxidation which further enhance the formation of reactive aldehydes. Levels of *gstr* mRNA from the flatfish plaice has been reported to be induced by β-naphthoflavone in intestine and spleen but not in gill, liver, brain, or testis [29]. Although this lab has reported that *gstr* is weakly induced by the electrophile cadmium in some tissues of adult coho [5] and herein confirm that the first 1800 bp of its promoter contains at least two potential AREs, this segment was unresponsive to any of the Nrf2s tested. It is possible a functional ARE may exist outside the segment assayed or that modulation may be independent of direct Nrf2 action. Nrf2-mediated expression has also been shown to be gene- and tissue-specific in a study of zebrafish larvae [38] indicating that ancillary factors may also be key components to the induction response; such factors would likely be absent from the *in vitro* cell model used in this study.

Zebrafish Nrf2 orthologs were included in reporter testing as a means to compare the previously unknown salmon forms to those of a model organism. *Dre*Nrf2a activation of the coho promoters was also contingent on the same AREs (Fig. 5, Fig. 6A). This zebrafish form also achieved slightly higher levels of activation than *Oki*Nrf2A and -B which might reflect a real difference in functionality or may simply be the result of greater compatibility with the cell line used (also derived from zebrafish). Previous work has shown that *gstp* in zebrafish is regulated by *Dre*Nrf2a through an ARE-like motif (TGACNNN*T*C) [47] and this may have influenced some studies to examine this ARE variant in zebrafish genome-wide [53]. However, since *Dre*Nrf2a will activate through salmon AREs that fit mammalian-derived consensus, it may be doubtful it would be limited to atypical sites in zebrafish. The second zebrafish form, *Dre*Nrf2b, has been shown to have the ability to act as a negative regulator in zebrafish embryos, including down-regulating the endogenous expression of *hmox1* and an exogenous zebrafish *gstp1* reporter [53]. In the embryonic cell line used in this study, however, *Dre*Nrf2b activated the salmon reporters of *gclc, gstp, hmox1*, and *prdx1,* albeit weakly (Fig. 5, Fig. 6A) in an ARE-dependent manner. If *Dre*Nrf2b then lacks intrinsic negative properties, it might instead complex with other unknown repressors available in whole embryos but either lacking or non-functional in the assayed cell line.

## 5 Conclusions

This work has examined one component of an underlying mechanism guiding oxidative response in a non-model organism, coho salmon. It has shown that like zebrafish, salmon have duplicate paralogs of Nrf2 but unlike zebrafish whose forms have underwent subfunctionalization, those of the salmon have both retained function as activators of transcription. Termed Nrf2A and Nrf2B, *in silico* analysis revealed high identity of virtually all residues known to be functionally critical. One of the few AREs previously delineated in teleosts was that of *gstp* in zebrafish. Varying from the canonical motif described from mammals (TGACNNN*T*C), it was uncertain if this one motif was anomalous or if it might be the prevalent form in fishes. This study delineates the functionality of AREs from four salmon genes, including *gstp, gclc, prdx1,* and *hmox1,* in an *in vitro* assay. All functional salmon AREs conformed to the mammalian consensus (RTGAYNNNGC). By contrast, functionality of AREs within the promoter of *gstr*, was not evident, possibly suggesting a function in general cell maintenance. This work should provide support to future examinations of mechanisms of Nrf2 and the ARE in teleosts and broadens its understanding for all vertebrate species, and may have implications for studies of Nrf2 target gene biomarkers in fish and their response to environmental chemicals in aquatic habitats.

## Appendix A Supplementary material

Supplementary material: Fig. 9. ***Supplemental.*****A serine at position 40 is absent from Nrf2 orthologs of many fish orders.** Alignment of the DLGex motif within the Nrf2 Neh2 domain among 16 fish species from 10 different orders and the human. DLGex is one half of the contact area needed to interact with the Nrf2 negative regulator Keap1. Shared positions are shaded. The serine site proposed to be phosphorylated in mammalian forms (box) is absent in teleosts from many orders. DLGex motif underscored. *Paralogs described in this study.

.

Supplementary material: Fig. 10. ***S******upplemental.*****A comparison of reporters used in this study.** Relative constitutive expression of the promoter reporters used in this study above background (pGL3 -, the empty vector), upward graph; numbers are averaged over three or more independent experiments. Fold induction of each wild-type reporter by coho Nrf2A or -2B relative to the constitutive levels of each, lower graph.
