# Supplementary material for: Dual NRF2 paralogs in Coho salmon and their antioxidant response element targets
Source: Redox Biol. 2016 Jul 6;9:114–23. doi: 10.1016/j.redox.2016.07.001 (PMC5068245; doi:10.1016/j.redox.2016.07.001)
Supplement: Supplementary file 1 — Supplementary material: Fig. 9. Supplemental.A serine at position 40 is absent from Nrf2 orthologs of many fish orders. Alignment of the DLGex motif within the Nrf2 Neh2 domain among 16 fish species from 10 different orders and the human. DLGex is one half of the contact area needed to interact with the Nrf2 negative regulator Keap1. Shared positions are shaded. The serine site proposed to be phosphorylated in mammalian forms (box) is absent in teleosts from many orders. DLGex motif underscored. *Paralogs described in this study. [file mmc1.pptx]

## Slide 1
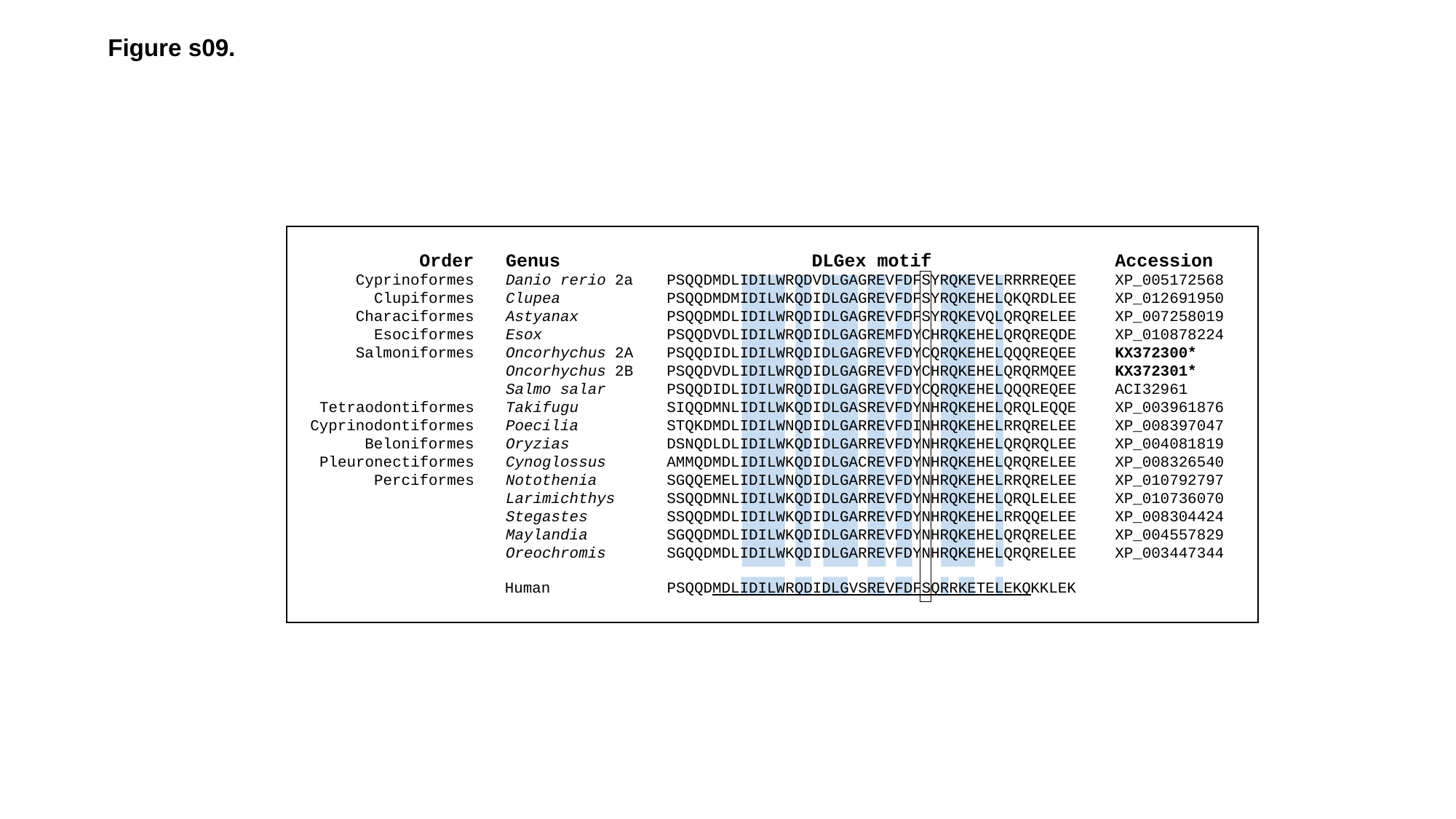

Figure s09.
Order
Cyprinoformes
Clupiformes
Characiformes
Esociformes
Salmoniformes
Tetraodontiformes
Cyprinodontiformes
Beloniformes
Pleuronectiformes
Perciformes
Genus
Danio rerio 2a Clupea
Astyanax
Esox
Oncorhychus 2A Oncorhychus 2B
Salmo salar
Takifugu
Poecilia
Oryzias
Cynoglossus
Notothenia
Larimichthys
Stegastes
Maylandia
Oreochromis
DLGex motif
PSQQDMDLIDILWRQDVDLGAGREVFDFSYRQKEVELRRRREQEE
PSQQDMDMIDILWKQDIDLGAGREVFDFSYRQKEHELQKQRDLEE
PSQQDMDLIDILWRQDIDLGAGREVFDFSYRQKEVQLQRQRELEE
PSQQDVDLIDILWRQDIDLGAGREMFDYCHRQKEHELQRQREQDE
PSQQDIDLIDILWRQDIDLGAGREVFDYCQRQKEHELQQQREQEE
PSQQDVDLIDILWRQDIDLGAGREVFDYCHRQKEHELQRQRMQEE
PSQQDIDLIDILWRQDIDLGAGREVFDYCQRQKEHELQQQREQEE
SIQQDMNLIDILWKQDIDLGASREVFDYNHRQKEHELQRQLEQQE
STQKDMDLIDILWNQDIDLGARREVFDINHRQKEHELRRQRELEE
DSNQDLDLIDILWKQDIDLGARREVFDYNHRQKEHELQRQRQLEE
AMMQDMDLIDILWKQDIDLGACREVFDYNHRQKEHELQRQRELEE
SGQQEMELIDILWNQDIDLGARREVFDYNHRQKEHELRRQRELEE
SSQQDMNLIDILWKQDIDLGARREVFDYNHRQKEHELQRQLELEE
SSQQDMDLIDILWKQDIDLGARREVFDYNHRQKEHELRRQQELEE
SGQQDMDLIDILWKQDIDLGARREVFDYNHRQKEHELQRQRELEE
SGQQDMDLIDILWKQDIDLGARREVFDYNHRQKEHELQRQRELEE
Accession
XP_005172568
XP_012691950
XP_007258019
XP_010878224
KX372300*
KX372301*
ACI32961
XP_003961876
XP_008397047
XP_004081819
XP_008326540
XP_010792797
XP_010736070
XP_008304424
XP_004557829
XP_003447344
Human
PSQQDMDLIDILWRQDIDLGVSREVFDFSQRRKETELEKQKKLEK

## Slide 2
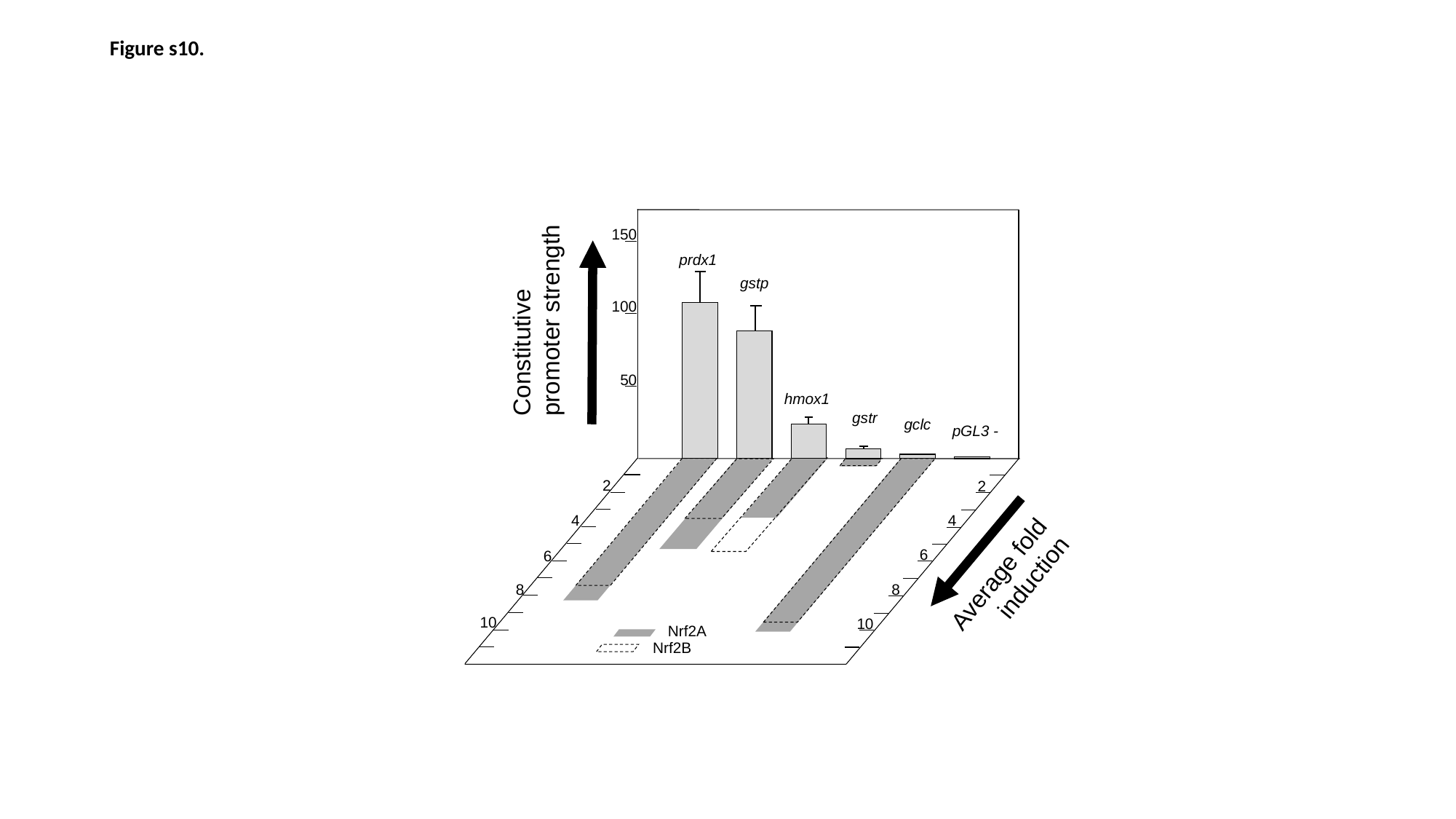

Figure s10.
150
prdx1
gstp
Constitutive promoter strength
100
50
hmox1
gstr
gclc
pGL3 -
2
2
4
4
6
6
8
8
Average fold induction
10
10
Nrf2A
Nrf2B
